# Campaign contributions and policy divergence

**DOI:** 10.1007/s00355-025-01601-1

**Published:** 2025-05-24

**Authors:** Tsz-Ning Wong, Nikolay Marinov

**Affiliations:** 1 Department of Economics and Barcelona Economic Analysis Team (BEAT), Universitat de Barcelona, Barcelona, Spain; 2 Department of Political Science, University of Gothenburg, Gothenburg, Sweden

## Abstract

Standard spatial models of political competition predict that competing candidates would converge to the same policy position. In this paper, we present a novel mechanism of policy divergence in a model of campaign finance, where campaign contributions from ideologically extreme donors can be used to boost the turnout of impressionable voters. In our model, the exact location of the median voter is unknown to the candidates. Together with donors’ contributions, this generates policy divergence even when the candidates are ex-ante identical and purely office-motivated. We characterize the unique equilibrium of the model in mixed strategies and provide closed form expression for the expected level of policy polarization. We further show that polarization is higher when only the incumbent can benefit from campaign contributions.

## Introduction

There is by now a widespread consensus that the major political parties in the United States have become more polarized over the past 40 years (Layman et al. 2006). Various mechanisms have been proposed to explain this trend. Some suggest that it simply reflects the ideological polarization among the American mass public (Abramowitz and Saunders 2008).^1^ Others suggest that ideological donors exerts disproportionate influence on the political parties and drive their policies away from the median voter in different directions (Verba 1995; Miller and Schofield 2003). The latter theory, however, faces challenges from both the theoretical and empirical sides.

On the theoretical side, it has been suggested that one should not expect policy polarization even in the presence of extreme donors (Hirata and Kamada 2020). Although the median voter theorem (Downs 1957), which predicts that candidates’ policies will converge to the median voter’s ideal point, applies only when ideological donors are absent, a classic result in the literature of campaign finance states that in the presence of a donor, the candidates will choose a policy that maximizes a weighted sum of the utilities of the voters and the donor (Grossman and Helpman 1996; Persson and Tabellini 2002). Therefore, barring heterogeneity in the candidates’ characteristics, we would expect that the candidates will adopt the same policy.^2^

On the empirical side, La Raja and Wiltse (2012) find that there is substantial ideological stability in the donor population in the United States until 2002 and that ideological citizens are not more likely to donate today than similarly ideological citizens in the past. As a result, the increasing trend of partisan polarization cannot be attributed to a more ideologically extreme donor population. Moreover, they find that the proportion of ideological donors varies from election to election with no discernible trend over time. They suggest that this is because “*strategic* politicians mobilize latent ideological elements of the donor population when it suits their needs” and that “the direction of causation appears more likely to run from politicians to donors.” Focusing on small donors, Malbin (2013) and Keena and Knight-Finley (2019) likewise reject the claim that small donors are a source of policy polarization.

In this paper, we contribute to this debate by presenting a Downsian model that generates policy divergence and sheds light on the strategic behavior suggested by La Raja and Wiltse (2012). Our model is a variant of the standard Downsian model with campaign contributions (Persson and Tabellini 2002), in which office-motivated candidates propose policies, an ideologically extreme donor decides whom to support and how much to contribute, and voters elect their representative. There are two groups of voters. *Informed* voters have single-peaked preferences on the policies and vote sincerely. *Uninformed* voters, on the other hand, have no preference on the policies but can be influenced by campaign spending.^3^ We assume that informed voters are uniformly distributed and the location of the median voter’s ideal point is also uncertain and uniformly distributed.

We first show that when the donor’s stake is high enough, a candidate always has the incentive to differentiate themselves from their opponent. As a result, an equilibrium in pure strategies does not exist. The reason is that the donor’s incentive to contribute is proportional to the distance between the candidates’ policies. This encourages opportunistic behavior from the candidates. Facing a centrist, a candidate has the incentive to adopt an extreme (pro-donor) position to maximize the donor’s contributions. Facing an extremist, a candidate has the incentive to adopt a slightly more moderate position than their opponent to appeal to the informed voters. Competition for the informed voters then pushes the candidates toward the center again, until at some point one of the candidates again finds it beneficial to adopt an extreme position. This means that the best response of the candidates is cyclical and, as a result, a pure strategy equilibrium does not exist.^4^

We then characterize the unique equilibrium in mixed strategies and quantify the expected level of policy polarization. We obtain a closed-form expression for polarization and show that it is single-peaked in the donor’s benefit-to-cost ratio to contribute, which is defined as the ratio of the donor’s stake and the amount of voter uncertainty. Our model thus suggests that campaign contributions can be linked to policy polarization in subtle ways. First, polarization is determined by voter preference uncertainty as well as the donor’s characteristics. A change in voter preference uncertainty, for example, can alter the donor’s cost-benefit calculus to contribute and give rise to polarization, even when the donor’s characteristics remain the same. Second, even though polarization is linked to the donor’s incentive to contribute, the relationship is not monotonic. Expected polarization first rises and then declines with the donor’s benefit-to-cost ratio. Finally, since the candidates randomize over their choices of policy in equilibrium, the relationship between policy polarization and political contributions is not deterministic and can be difficult to uncover empirically. All these can make the empirical study of polarization in the shadow of political donation tricky.^5^

Next, we consider an extension of the model in which only the incumbent can receive campaign contributions. In equilibrium the opposition is more likely to adopt a “moderate” policy in the sense that it represents a compromise between the voters’ and the donors’ preferences. On the other hand, the incumbent is more likely to adopt “extreme” policies that are either close to the expected median voter or to the donor. We show that this results in increased policy polarization comparing to the case when there is fair competition.

Finally, we show that our main results generalize to the multiple-donor case. In that case, the net effect of multiple donors can be summarized in an aggregated benefit-to-cost ratio and the equilibrium outcome of the game would be identical to one in which there is only a single donor with the corresponding benefit-to-cost ratio.

### Related literature

Our model belongs to the lobbying theory of political competition pioneered by Baron (1994) and Grossman and Helpman (1996), which incorporates campaign contributions by donors into the standard Downsian framework. Standard treatment of the problem (Persson and Tabellini 2002) assumes that the voters’ preferences are subject to a *valence* shock before voting. In equilibrium, the candidates choose the same policy and polarization does not occur.^6^ In contrast, in our model, the voters’ subjective evaluations of the *policies*, rather than the candidates, are random.^7^

Several approaches have been proposed in the literature to generate policy polarization in the presence of a donor. An overview of these approaches is given by Ursprung (2002).

One way of generating polarization is to change the objectives of the candidates. Glazer and Gradstein (2005) assume that, instead of maximizing the probability of winning, candidates aim to maximize the contributions they collect. This generates policy polarization in equilibrium, since, otherwise, both candidates would receive zero contribution and prefer to deviate. Alternatively, one can also assume that candidates are policy-motivated and have different policy preferences. Indeed, such a model can generate policy polarization even in the absence of a donor (Calvert 1985; Wittman 1983). To the best of our knowledge, the closest papers to ours are Ball (1999) and Dunaway and Munoz-Garcia (2020), who consider campaign contributions in such a setting. Ball (1999) shows that when the candidates are purely policy-motivated, increasing the effectiveness of donation from two symmetric donors with opposite interests *decreases* policy polarization in equilibrium.^8^ In contrast, we find that policy polarization first increases and then decreases. Dunaway and Munoz-Garcia (2020) allow asymmetric donors as well as a combination of policy and office motivations. However, they only provide numerical examples of pure strategy equilibria and simply note that when office motivation is high enough, a pure strategy equilibrium fails to exist. In contrast, our objective is to analyze the mixed strategy equilibrium with office-motivated candidates.

Another approach is to introduce additional assumptions or restrictions on players’ behaviors, while maintaining the assumption of office-motivated candidates. Hillman and Ursprung (1988) assume a protectionist candidate and a liberal trade policy candidate, who cater to different groups of donors. Similarly, Baron (1994) assumes that the candidates are aligned with different donor groups and generates policy divergence by considering competition in “particularistic” policy positions, where policy benefits to a donor can be denied if he has not contributed to the candidate. However, the policy divergence result disappears when competition in “collective” policy positions is considered instead, where the policy of the elected candidates affects all donors, regardless of their contributions, an assumption that we also maintain.

Other papers consider different formulations of the political game. Grossman and Helpman (1996) analyze a model with a sequence of moves different from ours, where the donors make contribution offers before the candidates choose policies. They show that there are two motives for campaign giving in their model: electoral motive (intention to affect a candidate’s probability of winning) and influence motive (intention to affect a candidate’s policy position). They show that when one of the candidates has a valence advantage over the other, the influence motive inclines the donor to contribute more to the front-runner and, as a result, creates a difference in the candidates’ policy positions, which can in turn prompt electorally motivated contributions. Rivas (2025) studies a variant of Grossman and Helpman (1996)’s model with two donors and the additional assumption that each donor can offer a contribution to only one candidate and each to a different candidate. He shows that polarization occurs in equilibrium even when no candidate has a valence advantage.

Our explanation for policy polarization complements these existing models. We maintain the assumption that the candidates are office-motivated and do not assume preference about policy, nor do we assume asymmetry in the candidates’ characteristics. In this sense, we trace the origin of policy divergence back to the candidate’s strategic incentives, rather than these other elements.

We also contribute more generally to the theoretical study of spatial models of political competition. Although a fair amount of work has been done to characterize mixed strategy equilibria in these models when pure strategy equilibria fail to exist, many works only provide partial characterizations of the mixed strategy equilibria (McKelvey 1986; Banks et al. 2002; Duggan 2007; Hummel 2010). In contrast, we show that our model admits a closed-form solution. Other works find mixed strategy equilibria that are very different from the one presented here (Aragonès and Xefteris 2012; Xefteris 2014).

The equilibrium construction in our paper is closely related to all-pay auction with complete information (Baye et al. 1993, 1996). In particular, Baye et al. (2012) solve for all symmetric Nash equilibria of a general, two-player, linear parameterized, symmetric rank-order contests with complete information. In contrast, we also consider an asymmetric game in which only the incumbent can receive campaign contributions. In the symmetric game where both candidates can receive campaign contributions, we also prove the uniqueness of equilibrium in addition to characterizing the equilibrium.

The rest of the paper is organized as follows. Section 2 introduces the model. Section 3 characterizes the unique equilibrium of the model and quantifies the expected level of polarization. Section 4 discusses the case when only the incumbent can receive contributions and shows that polarization increases as a result. Section 5 discusses the extension to include multiple donors. Section 6 concludes and suggests some directions for future research. Most of the proofs are relegated to the Appendix.

## Model

In our model, there are three groups of players: candidates, donors, and voters. We discuss them in turns.

**Candidates.** Two candidates, the incumbent *I* and the opposition *O*,  run for office in an election. In the baseline model, the two candidates are identical. In Sect. 4, we allow the incumbent to have an advantage over the opposition. The candidates simultaneously choose policies $x_{I}, x_{O}\in \left[ -1,1\right] .$ Note that the set $\left[ -1,1\right]$ does not necessarily represent the traditional left-right political spectrum. Instead, we assume that it represents a policy dimension that the donors care about. The objective of a candidate is to maximize their probability of winning the election.

**Donors.** After the candidates have chosen their policies, a donor chooses how much funding to contribute to the two candidates. To save notations, we assume in our baseline model that there is only a single donor. In Sect. 5, we show that our results generalize to the multiple-donor case. Given our timing of events, the donor will only contribute to one of the two candidates. Therefore, we assume, without loss of generality, that the donor chooses contribution $y\in \mathbb {R} ,$ where a positive *y* is interpreted as contributions for the incumbent *I* and a negative *y* is interpreted as contributions for the opposition *O*. The payoff function of the donor is^9^

$$\begin{aligned} u_{D}\left( y,x_{I},x_{O}\right)&=\psi \left[ \Pr \left( \,{\text {incumbent wins}}\,\right) x_{I}+\Pr \left( \,{\text {opposition wins}}\,\right) x_{O}\right] -\frac{y^{2}}{2}\\&=\psi x_{O}+\psi \Pr \left( \,{\text {incumbent wins}}\,\right) \left( x_{I}-x_{O}\right) -\frac{y^{2}}{2}. \end{aligned}$$

The parameter $\psi$ reflects the donor’s stake. The magnitude $\left| \psi \right|$ represents how important the elected candidate’s policy is to the donor. The sign of $\psi$ represents the donor’s ideal policy. If $\psi >0,$ the donor’s ideal policy is 1. If $\psi <0,$ the donor’s ideal policy is $-1.$^10^

**Voters.** Following Baron (1994) and Grossman and Helpman (1996), we assume that there are two groups of voters: the informed and uninformed. Informed voters are voters who care about a candidate’s policy. Uninformed voters, on the other hand, are indifferent about candidates’ policies, but ‘impressionable’ by campaign spending. We discuss them in turns.

There is a continuum of measure $2\phi$ of informed voters. They are uniformly distributed on the interval $\left[ \tilde{x}-\phi ,\tilde{x}+\phi \right] ,$ where $\tilde{x}$ is the position of the median voter. $\tilde{x}$ is itself a random variable that is uniformly distributed on $\left[ -\frac{\eta }{4},\frac{\eta }{4}\right] .$ Importantly, the realization of $\tilde{x}$ is unknown to the candidates and the donor. The random variable $\tilde{x}$ thus represents randomness in the election that is outside of the control of the candidates and the donor. One way to think about $\tilde{x}$ is an aggregate shock that shifts the preferences of the entire electorate.^11^ Informed voter *i* with ideal policy $x_{i}$ has preference that is single-peaked and symmetric around $x_{i}.$ We assume that informed voters vote sincerely and that indifferent voters vote for both candidates with equal probability.

Uninformed voters, on the other hand, are assumed to be uninterested in the policy and would otherwise abstain from voting. However, by spending the donor’s contribution $\left| y\right| ,$ a candidate can attract a measure $\left| y\right|$ of these voters to vote for them. We assume that the size of uninformed voters is larger than the size of informed voters so that it is always possible to win a election by spending enough on the campaign.^12^

In addition, we impose two assumptions on the parameters: $\phi >1+\frac{\eta }{4}$ and $4\left| \psi \right| <\eta \left( \eta -4\right) .$ The first assumption implies that in the absence of campaign contributions both candidates receive non-zero votes regardless of the policy choices $x_{I}$ and $x_{O}$ and the realization of $\tilde{x}.$ The second assumption ensures that the solution to the donor’s problem is interior, which we discuss in more detail later. Moreover, since $\left| \psi \right| \ge 0,$ it implies $\eta > 4,$ so that in the absence of campaign contributions both candidates win with non-zero probability regardless of the policy choices $x_{I}$ and $x_{O}.$

To summarize, the timing of events is as follows:

1. Two candidates simultaneously announce their policies $x_{I}$ and $x_{O}.$
2. The donor observes the chosen policies $x_{I}$ and $x_{O}$ and chooses *y*,  the *net* contribution to the incumbent *I*.
3. The voters vote simultaneously after observing $x_{I},$ $x_{O}.$
4. The payoffs are realized.

We look for subgame perfect equilibrium. We use $x_{I}^{*},$ $x_{O}^{*}$ and $y^{*}\left( x_{I},x_{O}\right)$ to denote the equilibrium choices of the incumbent, the opposition and the donor, respectively.

## Equilibrium analysis

To solve for the subgame perfect equilibrium of the election game, we work backward from the choice of the voters. Suppose $x_{I}>x_{O}.$ For a given $\tilde{x},$ the vote margin of the incumbent over the opposition is given by

$$ \underset{\,{\text {Incumbent's informed votes}}\,}{\underbrace{\left( \tilde{x} +\phi -\frac{x_{I}+x_{O}}{2}\right) }}-\underset{\,{\text {Opposition's informed votes}}\,}{\underbrace{\left( \frac{x_{I}+x_{O}}{2}-\left( \tilde{x} -\phi \right) \right) }}+y=2\tilde{x}-\left( x_{I}+x_{O}\right) +y. $$

**Fig. 1:**
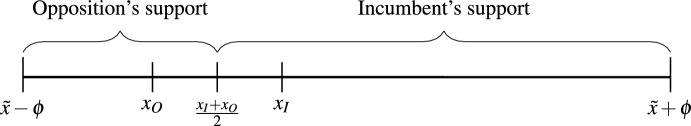
The candidates’ informed vote shares ($x_{I}>x_{O}$)

Figure 1 illustrates the candidates’ informed vote shares when $x_{I}>x_{O}.$

Similarly, if $x_{I}<x_{O},$ the vote margin is given by

$$ \left( \frac{x_{I}+x_{O}}{2}-\left( \tilde{x}-\phi \right) \right) -\left( \tilde{x}+\phi -\frac{x_{I}+x_{O}}{2}\right) +y=-2\tilde{x} +x_{I}+x_{O}+y. $$

Let $N_{y}\left( x_{I},x_{O}\right)$ denote the interval such that for a given $x_{I}$ and $x_{O},$ if the donor’s contribution *y* falls within $N_{y}\left( x_{I},x_{O}\right) ,$ then both candidates win with nonzero probability.^13^ Since $\tilde{x}$ is uniformly distributed on $\left[ -\frac{\eta }{4},\frac{\eta }{4}\right] ,$ given that $y\in N_{y}\left( x_{I},x_{O}\right) ,$ the probability that the incumbent wins is given by

1 $$\begin{aligned} \Pr \left( \,{\text {incumbent wins}}\,\right) =\left\{ \begin{array}{cc} \frac{1}{2}-\frac{x_{I}+x_{O}}{\eta }+\frac{y}{\eta } & \quad \,{\text {if }}\,x_{I} >x_{O},\\ \frac{1}{2}+\frac{x_{I}+x_{O}}{\eta }+\frac{y}{\eta } & \quad \,{\text {if }}\,x_{I} <x_{O}. \end{array} \right. \end{aligned}$$

Next, we solve the donor’s problem. Suppose $y\in N_{y}\left( x_{I},x_{O}\right) .$ The donor’s utility function can be written as

$$ u_{D}\left( y,x_{I},x_{O}\right) =\left\{ \begin{array}{cc} \psi x_{O}+\psi \left( \frac{1}{2}-\frac{x_{I}+x_{O}}{\eta }+\frac{y}{\eta }\right) \left( x_{I}-x_{O}\right) -\frac{y^{2}}{2} & \quad \,{\text {if }}\,x_{I}>x_{O},\\ \psi x_{O}-\frac{y^{2}}{2} & \quad \,{\text {if }}\,x_{I}=x_{O},\\ \psi x_{O}+\psi \left( \frac{1}{2}+\frac{x_{I}+x_{O}}{\eta }+\frac{y}{\eta }\right) \left( x_{I}-x_{O}\right) -\frac{y^{2}}{2} & \quad \,{\text {if }}\,x_{I}<x_{O}. \end{array} \right. $$

The solution to the donor’s problem is given by

2 $$\begin{aligned} y^{*}\left( x_{I},x_{O}\right) =\frac{\psi }{\eta }\left( x_{I}-x_{O}\right) . \end{aligned}$$

Thus, $y^{*}\left( x_{I},x_{O}\right)$ is interior, i.e., $y^{*}\left( x_{I},x_{O}\right) \in N_{y}\left( x_{I},x_{O}\right) .$ This is guaranteed by our second parametric assumption, which implies that $\frac{\left| \psi \right| }{\eta }\left( x_{I}-x_{O}\right) <x_{I}+x_{O}+\frac{\eta }{2}$ for all $x_{I},x_{O}\in \left[ -1,1\right] .$ Define a new parameter $\Psi$ by

$$ \Psi :=\frac{\psi }{\eta }, $$

which will be the key parameter for the rest of our analysis. $\Psi$ can be interpreted as the donor’s benefit-to-cost ratio for campaign contributions. A greater value signifies an election that is easier and more worthwhile to influence.

Our first result shows that when the donor’s interest is small, the median voter theorem (Hotelling 1929; Downs 1957) remains valid with campaign contributions.^14^

### Proposition 1

Suppose $\left| \Psi \right| <1.$ Then,  the median voter equilibrium remains the unique equilibrium of the election game with campaign contributions,  i.e.,  $x_{I}^{*}=x_{O}^{*}=0.$

This proposition nests the classic median voter theorem. When $\Psi =0$ (or, equivalently, when there is no donor), office-motivated candidates must locate at 0,  the ex-ante median of the voter ideal point distribution in equilibrium. To see why the median voter equilibrium survives in the election game with campaign contributions when $\left| \Psi \right| <1,$ suppose $x_{I}^{*}=x_{O}^{*}=0.$ If one of the candidates deviates to a point $x>0,$ they gain $\Psi x$ uninformed votes by using the donor’s campaign contribution. However, they also lose half of the informed votes between 0 and *x* to the other candidates. (The net loss of the informed votes is thus *x*.) Therefore, such a deviation will not be profitable if $\Psi \le 1.$ Similarly, a move to the left will not be profitable if $\Psi \ge -1.$ In the Appendix, we further show that there is no other equilibrium in this game when $\left| \Psi \right| <1.$

Next, suppose we have $\left| \Psi \right| >1.$ Our first observation is that a pure strategy equilibrium no longer exists.

### Lemma 1

Suppose $\left| \Psi \right| >1.$ Then,  the election game with campaign contributions does not have an equilibrium in pure strategies.

To see why this is the case, suppose $\left| \Psi \right| >1$ and an equilibrium in pure strategies exists. In equilibrium, both candidates must win with probability 1/2. This is because a candidate can guarantee themselves a 1/2 probability of winning by moving to their opponent’s position. This further implies that both candidates must locate themselves at 0,  because, if one of the candidates chooses a position $x^{*} \ne 0,$ then their opponent can move to point slightly to the left or right to their position (depending on the sign of $x^{*}$) and wins with a probability strictly larger than 1/2. But $x_{I}^{*}=x_{O}^{*}=0$ cannot be an equilibrium either. As we have seen in the discussion following Proposition 1, by moving to the donor’s ideal point, a candidate would gain $\left| \Psi \right|$ uninformed votes and lose only 1/2 informed votes to the opponent. Since $\left| \Psi \right| >1,$ this is a profitable deviation and, as a result, there is no pure strategy equilibrium.

Next, we proceed to find the unique equilibrium through the help of two lemmas. We focus on $\left| \Psi \right| >1$ case. The $\left| \Psi \right| <-1$ case is similar.

### Lemma 2

Suppose $\Psi >1.$ Then,  in any equilibrium of the election game with campaign contributions,  the support sets of the strategies must be an identical interval containing 1. Moreover,  the candidates’ equilibrium strategies contain no atom anywhere except at 1.

To understand the logic behind Lemma 2, notice first that since $\Psi >1,$ the candidates have no incentive to choose any point to the left of 0. Fixing candidate *j*’s policy at $x_{j}^{*} > 0,$ given (1) and (2), we can write candidate *i*’s payoff from choosing policy $x_{i}$ as

3 $$\begin{aligned} \Pr \left( \,{\text {candidate }}\,i\,{\text { wins}}\,\right) =\left\{ \begin{array}{cc} \left( \frac{1}{2}-\frac{\Psi }{\eta }x_{j}^{*}\right) -\frac{x_{j}^{*} }{\eta }+\frac{\Psi -1}{\eta }x_{i} & \quad \,{\text {if }}\,x_{i}>x_{j}^{*},\\ \left( \frac{1}{2}-\frac{\Psi }{\eta }x_{j}^{*}\right) +\frac{x_{j}^{*} }{\eta }+\frac{\Psi +1}{\eta }x_{i} & \quad \,{\text {if }}\,x_{i}<x_{j}^{*}. \end{array} \right. \end{aligned}$$

Note that the first term $\frac{1}{2}-\frac{\Psi }{\eta }x_{j}^{*}$ is independent of $x_{i},$ the second term changes sign when $x_{i}$ increases beyond $x_{j}^{*}$ and the third term strictly increases with $x_{i}$ on both sides of $x_{j}^{*}$ (however, it jumps downward at $x_{j}^{*}$). This means that if candidate *i* is indifferent between two points, candidate *j* must have assigned some probability mass in-between. Otherwise, the first and second terms remain constant, candidate *i* would strictly prefer the larger point because of the third term.^15^ As a result, if there is a gap $\left( x^{\prime },x^{\prime \prime }\right)$ in candidate *j*’s support set, then candidate *i* would not choose any of the points in $[x^{\prime },x^{\prime \prime }).$ But if candidate *i* would not choose any point in $[x^{\prime },x^{\prime \prime }),$ neither would candidate *j* choose $x^{\prime }.$ This implies that the interval $\left( x^{\prime },x^{\prime \prime }\right)$ must extend to include $-1.$ Thus, candidate *i*’s support set is an interval containing 1. Similar arguments show that the two intervals must be identical and the candidates do not assign any mass point in the support set except at 1.

### Lemma 3

Suppose $\Psi >1.$ Then,  in any equilibrium of the election game with campaign contributions,  candidate *i*’s strategy $F_{i}$ is differentiable and satisfies the differential equation

4 $$\begin{aligned} 2xf_{i}\left( x\right) +F_{i}\left( x\right) =\frac{\Psi +1}{2} \end{aligned}$$

in the interior of the support set,  where $f_{i}$ is the derivative of $F_{i}.$

In Lemma 2, we show that $F_{i}$ contains no atom in the interior of the support set. That is, $F_{i}$ is continuous. In the proof of Lemma 3 in the Appendix, we further show that $F_{i}$ is differentiable. Given that, (4) can be obtained from candidate *j*’s indifference condition by differentiating the objective function.

Finally, we claim that only one of the candidates can place an atom at 1. This is because, otherwise, one of the candidates can move the mass to a point slightly left to 1 and win with a strictly higher probability when the opponent chooses 1. This further implies that no candidate places a mass point at 1 and the equilibrium is symmetric, because (4) and the support sets are identical for both candidates and cannot admit two different solutions. Solving (4) explicitly, we have,

### Proposition 2

Suppose $\Psi >1.$ Then,  in the unique equilibrium of the election game with campaign contributions,  the candidates mix according to the distribution function

5 $$\begin{aligned} F\left( x\right) =\left\{ \begin{array}{cc} 0 & \quad \,{\text {if }}\,x\in [-1,\underline{x}^{*}),\\ \frac{\Psi +1}{2}-\frac{\Psi -1}{2}\frac{1}{\sqrt{x}} & \quad \,{\text {if }}\,x\in \left[ \underline{x}^{*},1\right] , \end{array} \right. \end{aligned}$$

where

6 $$\begin{aligned} \underline{x}^{*}:=\left( \frac{\Psi -1}{\Psi +1}\right) ^{2}. \end{aligned}$$

Similarly,  suppose $\Psi <-1.$ Then,  in the unique symmetric equilibrium of the election game with campaign contributions,  the candidates mix according to the distribution function

7 $$\begin{aligned} F\left( x\right) =\left\{ \begin{array}{cc} \frac{1+\Psi }{2}-\frac{1+\Psi }{2}\frac{1}{\sqrt{-x}} & \quad \,{\text {if }}\,x\in \left[ -1,\overline{x}^{*}\right] ,\\ 1 & \quad \,{\text {if }}\,x\in (\overline{x}^{*},1], \end{array} \right. \end{aligned}$$

where

8 $$\begin{aligned} \overline{x}^{*}:=-\left( \frac{1+\Psi }{1-\Psi }\right) ^{2}. \end{aligned}$$

The nonexistence of a pure strategy equilibrium is a result of the fact that the candidates are serving two masters. Depending on what the other candidate does, a candidate sometimes wants to appease the donor and sometimes wants to appeal to the voters.

It is straightforward to show an increase in $\Psi$ stochastically moves the candidates’ chosen policies toward the donor’s ideal point.

### Proposition 3

In the equilibrium of the election game with campaign contributions,  the candidates’ policy choices shift to the right in the sense of first-order stochastic dominance as $\Psi$ increases.

**Fig. 2:**
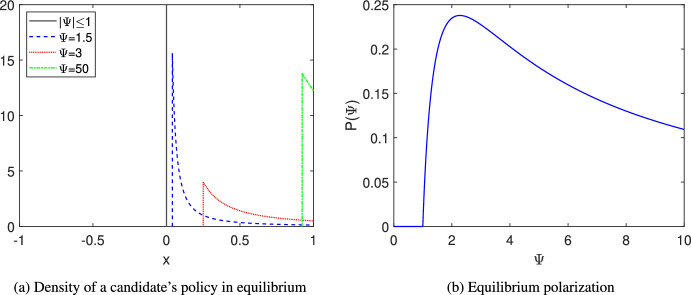
Illustrations of the Single-Donor Case

Figure 2a displays equilibrium policy densities. Since the two candidates use the same strategy in equilibrium, it is enough to illustrate the density for one of the candidates. As $\Psi$ increases, the distribution *F* shifts to the right. However, as $\Psi$ increases from 1.5 to 3,  the equilibrium policies become more dispersed, while as $\Psi$ increases from 3 to 50,  the equilibrium policies become more concentrated. We can quantify this change by considering the *equilibrium polarization*.

When the candidates use pure strategies in equilibrium, polarization can be understood as the distance between their chosen policies, i.e., $\left| x_{I}^{*}-x_{O}^{*}\right| .$ When the candidates use mixed strategies, we can understand polarization in terms of the expected distance. Thus, we define polarization *P* by

$$ P:={\mathbb {E}}\left( \left| x_{I}^{*}-x_{O}^{*}\right| \right) . $$

The closed form solution (5)–(8) of the mixed strategy equilibrium allows us to obtain an expression of polarization *P*.

### Proposition 4

The equilibrium polarization $P\left( \Psi \right)$ in the election game with campaign contributions is given by

9 $$\begin{aligned} P\left( \Psi \right) =\left\{ \begin{array}{cc} 0 & \quad \,{\text {if }}\,\left| \Psi \right| \le 1,\\ \left( \left| \Psi \right| -1\right) ^{2}\log \left( \frac{\left| \Psi \right| -1}{\left| \Psi \right| +1}\right) +2\left| \Psi \right| \left( \frac{\left| \Psi \right| -1}{\left| \Psi \right| +1}\right) & \quad \,{\text {if }}\,\left| \Psi \right| >1. \end{array} \right. \end{aligned}$$

Moreover,  $P\left( \Psi \right)$ is single-peaked for $\Psi \in [1,\infty )$ and satisfies $\lim _{\Psi \downarrow 1}P\left( \Psi \right) =\lim _{\Psi \uparrow +\infty }P\left( \Psi \right) =0.$

By Proposition 4, policy convergence occurs when the donor has either a very low or very high stake in the policy. In the former scenario, there is no campaign contributions in equilibrium and the policies converge to the ex-ante median point. In the latter scenario, the candidates compete for the donor’s support by placing themselves very close to the donor’s ideal point. As $\Psi$ approaches infinity, the candidates’ policies also approach the donor’s ideal point.

Figure 2b illustrates how equilibrium polarization *P* depends on $\Psi .$ The level of polarization is highest when the donor has an intermediate stake in the country. This is because candidates have incentives to cater to the median voter - but they are pulled aside by the donor’s contributions, which can also help secure support. When neither force predominates, positions diverge the most.

## One-sided contributions

In the previous sections, we assume that the donor can offer contributions to any one of the two candidates. In reality, the incumbent is likely to enjoy considerably incumbency advantage in fundraising (Fouirnaies and Hall 2014). Moreover, Culberson et al. (2019) find that incumbents raise more small donations by adopting extreme positions, while their opponents do not similarly benefit from extremism. In this section, we consider a setting in which only the incumbent can receive campaign contributions from the donor. The motivation for this is twofold. First, the incumbent can pass laws or use other administrative means to prevent the opposition from receiving contributions, while facing no such restrictions themselves (possibly because the law is applied unequally). This is especially likely to be the case in authoritarian countries, where elections are generally not “free and fair”. Second, the opposition may lack or at least not possess to the same degree as the government the political wherewithal to use the funding effectively to increase their vote tally. This also means that the donor will be less willing to offer support to the opposition. We consider here the extreme case where the opposition cannot make use of campaign contributions at all.

Mathematically, this means that the donor is restricted to choose $y\ge 0.$ We show that the incumbent chooses extreme policies in this setting. As a result, this increases the polarization of the candidates’ policies. We focus on the $\Psi \ge 0$ case. The $\Psi <0$ case is similar.

Again we solve the game by backward induction. It is easy to see that in this case the donor’s equilibrium contribution to the incumbent $y^{*}\left( x_{I},x_{O}\right)$ is given by

$$ y^{*}\left( x_{I},x_{O}\right) =\Psi \max \left\{ x_{I}-x_{O},0\right\} . $$

Proposition 5 shows that with one-sided contributions, the support sets of the two candidates’ strategies coincide as before. However, unlike the unrestricted contribution case, in this case the incumbent chooses the donor’s ideal policy 1 with non-zero probability. That is, the incumbent places an atom at 1.

### Proposition 5

Suppose only the incumbent *I* can receive contributions from the donor. If $0\le \Psi <1,$ then the median voter equilibrium remains the unique equilibrium of the election game with campaign contributions,  i.e.,  $x_{I}^{*}=x_{O}^{*}=0.$ If $\Psi >1,$ there exists an equilibrium in which the incumbent *I* mixes according to the distribution function

10 $$\begin{aligned} F_{I}\left( x\right) =\left\{ \begin{array}{cc} 0 & \quad \,{\text {if }}\,x\in [-1,\underline{x}^{**}),\\ \frac{1+\Psi }{2+\Psi }\left[ 1-\left( \Psi -1\right) ^{\frac{2+\Psi }{2-\Psi } }x^{-\frac{2+\Psi }{4}}\right] & \quad \,{\text {if }}\,x\in [\underline{x}^{**},1) \,{\text { and }}\,\Psi \ne 2,\\ \frac{3}{4}\left[ 1-e^{-4}x^{-1}\right] & \quad \,{\text {if }}\,x\in [\underline{x}^{**},1) \,{\text { and }}\,\Psi =2,\\ 1 & \quad \,{\text {if }}\,x=1, \end{array} \right. \end{aligned}$$

and the opposition *O* mixes according to the distribution function

11 $$\begin{aligned} F_{O}\left( x\right) =\left\{ \begin{array}{cc} 0 & \quad \,{\text {if }}\,x\in [-1,\underline{x}^{**}),\\ \frac{1}{2-\Psi }-\frac{\Psi -1}{2-\Psi }x^{-\frac{2-\Psi }{4}} & \quad \,{\text {if }}\, x\in \left[ \underline{x}^{**},1\right] \,{\text { and }}\,\Psi \ne 2,\\ 1+\frac{1}{4}\log \left( x\right) & \quad \,{\text {if }}\,x\in \left[ \underline{x} ^{**},1\right] \,{\text { and }}\,\Psi =2, \end{array} \right. \end{aligned}$$

where

12 $$\begin{aligned} \underline{x}^{**}:=\left\{ \begin{array}{cc} \left( \Psi -1\right) ^{\frac{4}{2-\Psi }} & \quad \,{\text {if }}\,\Psi \ne 2,\\ e^{-4} & \quad \,{\text {if }}\,\Psi =2. \end{array} \right. \end{aligned}$$

Note that the incumbent places an atom of size

$$ A_{I}:=\left\{ \begin{array}{cc} 1-\frac{1+\Psi }{2+\Psi }\left( 1-\left( \Psi -1\right) ^{\frac{2+\Psi }{2-\Psi }}\right) & \quad \,{\text {if }}\,\Psi \ne 2,\\ \frac{1}{4}+\frac{3}{4}e^{-4} & \quad \,{\text {if }}\,\Psi =2, \end{array} \right. $$

at the donor’s ideal policy 1. In contrast, in the symmetric case, the probability that a candidate chooses policy 1 is 0. In the Appendix, we prove Proposition 5 using the “guess and verify” approach.

Proposition 6 further compares the distributions $F_{I}$ and $F_{O}.$

### Proposition 6

Suppose $$\Psi >1.$$ Then,  there exists a unique $$\hat{x}\in \left( \underline{x}^{**},1\right) $$ such that for all $$x\in \left( \underline{x}^{**},\hat{x}\right) ,$$ $$F_{I}\left( x\right) >F_{O}\left( x\right) ,$$ and for all $$x\in \left( \hat{x},1\right) ,$$ $$F_{I}\left( x\right) <F_{O}\left( x\right) .$$ Moreover,  $$F_{I}\left( \hat{x}\right) =F_{O}\left( \hat{x}\right) >\frac{1}{2}.$$

**Fig. 3:**
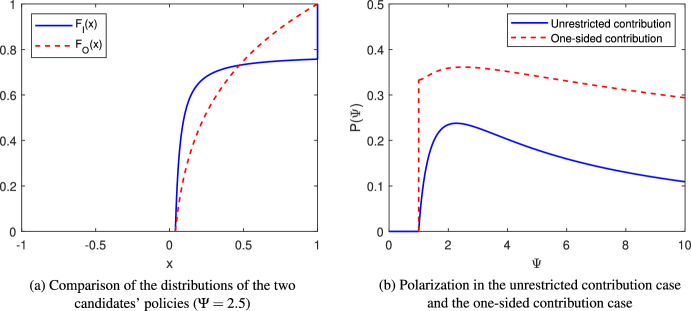
Illustrations of the one-sided contribution case

Proposition 6 suggests that in equilibrium the incumbent tends to choose extreme policies more than the opposition. That is, the incumbent puts an atom at the donor’s ideal policy at 1 and a larger probability mass near $$\underline{x}^{**},$$ the left end of the common support set. The opposition responses by choosing more moderate positions, i.e., points in the middle range of support set $$\left[ \underline{x}^{**},1\right] .$$ Figure 3a illustrates the two distributions when $$\Psi =2.5.$$

It is instructive to consider the limit equilibrium when $\Psi$ approaches 1 from above. In the limit, the incumbent chooses $x_{I}^{*}=0$ with probability $\frac{2}{3}$ and $x_{I}^{*}=1$ with probability $\frac{1}{3}$ and the opposition chooses $x_{O}^{*}=0$ with probability 1. Given that the donor always sets $y^{*}\left( x_{I},x_{O}\right) =\max \left\{ x_{I}-x_{O},0\right\}$ and the opposition chooses $x_{O}^{*}=0,$ by (1), the incumbent’s payoff of choosing any $x \ge 0$ is given by

$$ \Pr \left( \,{\text {incumbent wins}}\,\right) =\frac{1}{2}-\frac{x}{\eta }+\frac{y^{*}\left( x, 0\right) }{\eta }=\frac{1}{2}. $$

Thus, the incumbent is indifferent between policies 0 and 1. On the other hand, given the incumbent’s strategy, by (1), the opposition’s payoff of choosing any $x \in [0,1)$ is given by

$$\begin{aligned}&\Pr \left( \,{\text {opposition wins}}\,\right) \\&\quad =\Pr \left( x_{I}^{*}=0\right) \times \left( \frac{1}{2}-\frac{x}{\eta }\right) +\Pr \left( x_{I}^{*}=1\right) \times \left( \frac{1}{2}+\frac{1+x}{\eta }-\frac{1-x}{\eta }\right) \\&\quad =\frac{1}{2}-\frac{2}{3}\frac{x}{\eta }+\frac{1}{3}\frac{2x}{\eta }\\&\quad =\frac{1}{2}. \end{aligned}$$

Thus, the opposition is indifferent between any $x\in [0,1).$ In contrast to the unrestricted contribution case, the incumbent’s strategy changes discontinuously at $\Psi =1.$ This leads to an upward jump in equilibrium polarization $P\left( \Psi \right)$ at $\Psi =1.$

$$ \lim _{\Psi \downarrow 1}P\left( \Psi \right) =\Pr \left( x_{I}^{*}=1\right) \times \left( 1-0\right) =\frac{1}{3}>0, $$

whereas both candidates locate themselves at 1 when contributions are unrestricted. Thus, being able to use campaign contributions for reelection, while denying the opposition access to contributions, enables the incumbent to offer a position most out of sync with the popular mandate. This makes their policies more diverged. More generally, we can use (10)-(12) to compute $P\left( \Psi \right)$ explicitly.

### Proposition 7

Suppose only the incumbent *I* can receive contributions from the donor. When $0\le \Psi <1,$ the equilibrium polarization $P\left( \Psi \right)$ is equal to 0. When $\Psi \in \left( 1,\infty \right) \backslash \left\{ 2\right\} ,$ $P\left( \Psi \right)$ is given by

13 $$\begin{aligned} P\left( \Psi \right)&=\frac{\Psi ^{2}+2}{\left( \Psi +2\right) ^{2} }+\frac{8\left( \Psi +1\right) \left( \Psi -1\right) ^{\frac{4}{2-\Psi }} }{\left( \Psi -2\right) ^{2}\left( \Psi +2\right) }\log \left( \Psi -1\right) \nonumber \\&-\frac{\Psi ^{3}+5\Psi ^{2}+7\Psi +6}{\left( \Psi -2\right) \left( \Psi +2\right) ^{2}}\left( \Psi -1\right) ^{\frac{4}{2-\Psi }}-\frac{\Psi ^{3}+2\Psi ^{2}+11\Psi +10}{\left( \Psi -2\right) \left( \Psi +2\right) ^{2} }\left( \Psi -1\right) ^{\frac{2+\Psi }{2-\Psi }}. \end{aligned}$$

Moreover,  $$\lim _{\Psi \downarrow 1}P\left( \Psi \right) =\frac{1}{3} >0=\lim _{\Psi \uparrow 1}P\left( \Psi \right) $$ and $$\lim _{\Psi \uparrow +\infty }P\left( \Psi \right) =0.$$^16^

Figure 3b compares polarization in the unrestricted contribution case and the one-sided contribution case. In spite of the jump at $\Psi = 1,$ the equilibrium polarization $P\left( \Psi \right)$ remains single-peaked in $\Psi$ in the one-sided contribution case.^17^ However, the level of polarization increases when contributions are one-sided.^18^ Intuitively, by Proposition 6, in the one-sided contribution case, the incumbent chooses “extreme” points, i.e., points that are close to the two ends of the common support set, more often than the opposition. As a result, two distributions does not “overlap” as much and this leads to increased polarization.^19^

## Multiple donors

In the previous sections, we study the case of a single donor. However, in the American National Election Studies (ANES), roughly 10 percent of Americans report donating to a campaign.^20^ It is therefore of interest to see how our model extends to the multiple-donor case. Indeed, it does.

Suppose the players’ choices are such that both candidates win with nonzero probability, i.e., $y_{i}+\sum _{j\ne i}y_{j}^{*}\left( x_{I},x_{O}\right) \in N_{y}\left( x_{I},x_{O}\right) .$ Then, donor *i*’s utility function can be written as

$$\begin{aligned} &  u_{i}\left( y_{i},y_{-i}^{*}\left( x_{I},x_{O}\right) ,x_{I},x_{O}\right) \\ &  \qquad =\left\{ \begin{array}{cc} \psi _{i}x_{O}{+}\psi _{i}\left( \frac{1}{2}{-}\frac{x_{I}{+}x_{O} }{\eta }{+}\frac{y_{i}{+}\sum _{j\ne i}y_{j}^{*}\left( x_{I},x_{O}\right) }{\eta }\right) \left( x_{I}{-}x_{O}\right) -\frac{y_{i}^{2}}{2} & \quad \,{\text {if }}\,x_{I} >x_{O},\\ \psi _{i}x_{O}{-}\frac{y_{i}^{2}}{2} & \quad \,{\text {if }}\,x_{I}=x_{O},\\ \psi _{i}x_{O}{+}\psi _{i}\left( \frac{1}{2}{+}\frac{x_{I}{+}x_{O} }{\eta }{+}\frac{y_{i}+\sum _{j\ne i}y_{j}^{*}\left( x_{I},x_{O}\right) }{\eta }\right) \left( x_{I}{-}x_{O}\right) {-}\frac{y_{i}^{2}}{2} & \quad \,{\text {if }}\,x_{I} <x_{O}. \end{array} \right. \end{aligned}$$

It is easy to see that donor *i*’s equilibrium contribution to the incumbent $y_{i}^{*}\left( x_{I},x_{O}\right)$ remains given by

14 $$\begin{aligned} y_{i}^{*}\left( x_{I},x_{O}\right) =\frac{\psi _{i}}{\eta }\left( x_{I}-x_{O}\right) , \end{aligned}$$

as long as $4\left| \sum _{i=1}^{N}\psi _{i}\right| <\eta \left( \eta -4\right) .$ Define $\Psi _{i}:=\frac{\psi _{i}}{\eta }.$ The total effect of the donors’ contributions is simply

$$ \sum _{i=1}^{N}y_{i}^{*}\left( x_{I},x_{O}\right) =\sum _{i=1}^{N}\frac{\psi _{i}}{\eta }\left( x_{I}-x_{O}\right) =\left( \sum _{i=1}^{N}\Psi _{i}\right) \left( x_{I}-x_{O}\right) . $$

Therefore, we can simply define

$$ \Psi :=\sum _{i=1}^{N}\Psi _{i}. $$

And the previous analysis applies. The candidates’ equilibrium strategies are characterized by Proposition 2 while the choice of an individual donor can be obtained from (14). When the model is symmetric, the influences of opposite donors balance out, i.e., $\sum _{i=1}^{N}\psi _{i}=0,$ and the median voter theorem remains valid. This is demonstrated by Dunaway and Munoz-Garcia (2020). Our result further covers the case of asymmetric donors.

## Conclusion

In this paper, we study a novel mechanism through which candidates, running in elections, split on policies in the presence of political donors. We show that key factors for policy polarization are donors’ preference intensities, voters’ preference uncertainty, and restrictions on the opposition’s campaign finance. Our approach does not require pre-existing divisions in society or among the candidates on issues of importance to the donors.

However, our theory of policy divergence also has its limitations. One obvious objection to the equilibrium we obtain is that the two major political parties in the United States seem to be polarized in different directions. For example, in the abortion debate, the Republican Party is always deemed to be “pro-life” and the Democratic Party “pro-choice”. In contrast, in the equilibrium of our model, both parties would choose a certain position with the same probability. One way of explaining this is that, in reality, candidates are constrained by the traditional policy stance of their own party and, as a result, unable to choose policy freely as supposed in the model. In this sense, our model is best applied to situations where there is no pre-existing divisions among the parties.^21^

On the other hand, while it has been argued that party history and policy persistence can generate polarization in models of campaign finance, early work (Hillman and Ursprung 1988, 1993) presumes such a division in the candidates’ inherited party stances. One of the directions for future research, therefore, would be to introduce policy persistence (Forand 2014; Nunnari and Zápal 2017) formally in a multiple-period model with campaign contributions.

## Data Availability

Not applicable.

## Appendix

### Proof of Proposition 1

We have shown in the main text that $x_{I}^{*}=x_{O}^{*}=0$ is an equilibrium. We will now show that there is no other equilibrium. Suppose in equilibrium candidates *I* and *O* use (mixed) strategies $F_{I}$ and $F_{O},$ respectively. Suppose one of the candidates chooses policies on the right with nonzero probability. For $i\in \left\{ I,O\right\} ,$ let $\overline{x}_{i}:=\inf \left\{ x\in \left[ -1,1\right] :F_{i}\left( x\right) =1\right\} .$ We must have $\overline{x}_{I}=\overline{x}_{O}>0.$ If $\overline{x}_{i}>\overline{x}_{j},$ candidate *i*’s payoff for choosing $x^{\prime }\in (\overline{x}_{j},\overline{x}_{i}]$ is given by

$$\begin{aligned} \Pi _{i}\left( x^{\prime }\right)&=\int _{[-1,\overline{x}_{j}]}\left( \frac{1}{2}+\frac{-x^{\prime }-x+\Psi \left( x^{\prime }-x\right) }{\eta }\right) dF_{j}\left( x\right) \\&<\int _{[-1,\overline{x}_{j}]}\left( \frac{1}{2}+\frac{-x^{\prime \prime }-x+\Psi \left( x^{\prime \prime }-x\right) }{\eta }\right) dF_{j}\left( x\right) \\&=\Pi _{i}\left( x^{\prime \prime }\right) , \end{aligned}$$

where $x^{\prime \prime }\in (\overline{x}_{j},x^{\prime }).$ Thus, candidate *i* can improve his payoff by moving the probability mass on $(\overline{x}_{j},\overline{x}_{i}]$ toward $\overline{x}_{j}.$ Let $\overline{x}:=\overline{x}_{I}=\overline{x}_{O}.$ Note that the distributions $F_{I}$ and $F_{O}$ cannot both have a mass point at the same point at any $x \in (0,\overline{x}].$ Otherwise, a candidate can move the mass slightly to the left of *x* and receive a higher payoff. Suppose $F_{i}$ does not have a mass point at $\overline{x}.$ Since $F_{i}$ can have at most countably many jumps, for all $x^{\prime }\in \left( -1,\overline{x}\right)$ where $F_{i}$ does not jump, we have

$$\begin{aligned} \Pi _{j}\left( x^{\prime }\right) =\,&\int _{[-1,x^{\prime })}\left( \frac{1}{2}+\frac{-x^{\prime }-x+\Psi \left( x^{\prime }-x\right) }{\eta }\right) dF_{i}\left( x\right) \\&+\int _{(x^{\prime },\overline{x}]}\left( \frac{1}{2}+\frac{x^{\prime }+x+\Psi \left( x^{\prime }-x\right) }{\eta }\right) dF_{i}\left( x\right) . \end{aligned}$$

Thus, for all $x^{\prime \prime }\in \left( -1,x^{\prime }\right)$ where $F_{i}$ does not jump, we have

$$\begin{aligned} \Pi _{j}\left( x^{\prime \prime }\right) -\Pi _{j}\left( x^{\prime }\right) =&\int _{[-1,x^{\prime \prime })}\frac{\left( \Psi -1\right) \left( x^{\prime \prime }-x^{\prime }\right) }{\eta }dF_{i}\left( x\right) \\&+\int _{(x^{\prime \prime },x^{\prime })}\left( \frac{x^{\prime \prime }+x^{\prime }+2x+\Psi \left( x^{\prime \prime }-x^{\prime }\right) }{\eta }\right) dF_{i}\left( x\right) \\&+\int _{(x^{\prime },\overline{x}]}\frac{\left( \Psi +1\right) \left( x^{\prime \prime }-x^{\prime }\right) }{\eta }dF_{i}\left( x\right) \\ =&\left[ \frac{\Psi }{\eta }-\frac{1}{\eta }\int _{[-1,x^{\prime \prime })} dF_{i}\left( x\right) +\int _{(x^{\prime \prime },x^{\prime })}\left( \frac{x^{\prime \prime }+x^{\prime }+2x}{\eta \left( x^{\prime \prime }-x^{\prime }\right) }\right) dF_{i}\left( x\right) \right. \\&\left. +\frac{1}{\eta }\int _{(x^{\prime },\overline{x}]}dF_{i}\left( x\right) \right] \left( x^{\prime \prime }-x^{\prime }\right) . \end{aligned}$$

Since $\left| \Psi \right| <1,$ as $x^{\prime \prime },x^{\prime }\uparrow \overline{x},$ the term in the square bracket becomes strictly negative. This means that candidate *j* cannot place any probability mass near $\overline{x},$ which is a contradiction.

Similar arguments show that the candidates cannot choose policies on the left with nonzero probability. $\square$

### Proof of Lemma 1

In the main text. $\square$

### Proof of Lemma 2

Suppose in equilibrium candidates *I* and *O* use (mixed) strategies $F_{I}$ and $F_{O},$ respectively. Arguments similar to those in the proof of Proposition 1 show that $\lim _{x\uparrow 0}F_{I}\left( x\right) =\lim _{x\uparrow 0}F_{O}\left( x\right) =0.$

We next show that there cannot be any atom in $F_{I}$ and $F_{O},$ except at $x=0$ and $x=1.$ Suppose $F_{i}$ has an atom at $x^{\prime }\in \left( 0,1\right) .$ Then, candidate *j* would never choose a policy from an interval $[x^{\prime },x^{\prime \prime }),$ where $x^{\prime \prime }$ is larger than but close enough to $x^{\prime }.$ This is because candidate *j* can improve his payoff by moving to a point slightly to the left of $x^{\prime }$ instead. Next, let $\Pi _{i}\left( x\right)$ be candidate *i*’s expected payoff of choosing policy *x*. For any $x^{\prime \prime \prime }\in [x^{\prime },x^{\prime \prime }),$

$$\begin{aligned} \Pi _{i}\left( x^{\prime \prime \prime }\right) =&\int _{\left[ 0,x^{\prime }\right] }\left( \frac{1}{2}+\frac{-x^{\prime \prime \prime }-x+\Psi \left( x^{\prime \prime \prime }-x\right) }{\eta }\right) dF_{j}\left( x\right) \\&+\int _{\left[ x^{\prime \prime },1\right] }\left( \frac{1}{2}+\frac{x^{\prime \prime \prime }+x+\Psi \left( x^{\prime \prime \prime }-x\right) }{\eta }\right) dF_{j}\left( x\right) . \end{aligned}$$

Thus, for all $x\in [x^{\prime },x^{\prime \prime }),$

$$ \Pi _{i}^{\prime }\left( x\right) =\frac{1}{\eta }\left( \Psi +1-2F_{j}\left( x^{\prime }\right) \right) >0, $$

as $\Psi >1.$ Thus, candidate *i* strictly prefers $x\in \left( x^{\prime },x^{\prime \prime }\right)$ over $x^{\prime }.$ $F_{i}$ cannot have an atom at $x^{\prime }.$

Next, we rule out an atom at $x^{\prime }=0$ with a similar argument. Suppose $F_{i}\left( 0\right) >0.$ Then, candidate *j* would never choose a policy from an interval $\left( 0,x^{\prime \prime }\right) ,$ where $x^{\prime \prime }$ is larger than but close enough to 0. Unlike the previous case, we have not ruled out the possibility that candidate *j* may place an atom at 0 in this case. But it remains true that $\lim _{x\downarrow 0}\Pi _{i}\left( x\right) =\Pi _{i}\left( 0\right) .$ A similar calculation thus implies that for all $x\in \left( x^{\prime },x^{\prime \prime }\right) ,$ we again have $\Pi _{i}\left( x\right) >\Pi _{i}\left( 0\right) .$ Thus, $F_{i}$ cannot have an atom at 0 either.

The above argument can be used to further show that the support sets of the two distributions must be an identical interval containing 1. This is because whenever there is an interval $[x^{\prime },x^{\prime \prime } )\subseteq (0,1]$ on which the distribution $F_{j}$ does not put any probability mass, i.e., $\lim _{x\uparrow x^{\prime \prime }}F_{j}\left( x\right) -F_{j}\left( x^{\prime }\right) =0,$ we must have, for all $x\in \left( x^{\prime },x^{\prime \prime }\right) ,$ $\Pi _{i}^{\prime }\left( x\right) >0,$ so that candidate *i* would not use any $x\in [x^{\prime }-\delta ,x^{\prime \prime }),$ where $\delta >0$ is small enough. The identical argument then can be applied to candidate *j* to show that candidate *j* would never choose any $x\in [x^{\prime }-\varepsilon ,x^{\prime \prime }),$ where $\varepsilon >0$ is small enough. Applying this observation to different cases, we conclude that (i) the support sets of $F_{I}$ and $F_{O}$ must be intervals containing 1 (this case is discussed in the main text), and (ii) the intervals must be identical. $\square$

### Proof of Lemma 3

Candidate *j*’s expected payoff of choosing $x^{\prime } \in \left( 0,1\right)$ is given by

$$\begin{aligned} \Pi _{j}\left( x^{\prime }\right) =&\frac{\Psi }{\eta }x^{\prime }-\int _{(0,x^{\prime }]}\left( \frac{x^{\prime }+x}{\eta }\right) dF_{i}\left( x\right) \\&+\int _{(x^{\prime },1]}\left( \frac{x^{\prime }+x}{\eta }\right) dF_{i}\left( x\right) +\frac{1}{2} -\frac{\Psi }{\eta }{\mathbb {E}}\left( x\right) . \end{aligned}$$

For any $x^{\prime }$ and $x^{\prime \prime }$ in the interior of the support set of $F_{j},$ we must have

$$ \Pi _{j}\left( x^{\prime \prime }\right) -\Pi _{j}\left( x^{\prime }\right) =0. $$

Suppose $x^{\prime \prime }-x^{\prime }=\varepsilon >0.$ We have

$$\begin{aligned} 0 =&\frac{\Psi }{\eta }\varepsilon -\frac{\varepsilon }{\eta }F_{i}\left( x^{\prime }\right) +\frac{\varepsilon }{\eta }\left( 1-F_{i}\left( x^{\prime \prime }\right) \right) \\&-\int _{x^{\prime }}^{x^{\prime \prime }}\left( \frac{x^{\prime \prime }+x}{\eta }\right) dF_{i}\left( x\right) -\int _{x^{\prime }}^{x^{\prime \prime }}\left( \frac{x^{\prime }+x}{\eta }\right) dF_{i}\left( x\right) \\ =&\frac{\Psi +1}{\eta }\varepsilon -\frac{\left( x^{\prime \prime }-x^{\prime }\right) }{\eta }\left( F_{i}\left( x^{\prime }\right) +F_{i}\left( x^{\prime \prime }\right) \right) \\&-\frac{\left( x^{\prime \prime }+x^{\prime }\right) }{\eta }\left( F_{i}\left( x^{\prime \prime }\right) -F_{i}\left( x^{\prime }\right) \right) -2\int _{x^{\prime }}^{x^{\prime \prime }}\frac{x}{\eta }dF_{i}\left( x\right) \\ =&\frac{\Psi +1}{\eta }\varepsilon -\frac{2x^{\prime \prime }F_{i}\left( x^{\prime \prime }\right) }{\eta }+\frac{2x^{\prime }F_{i}\left( x^{\prime }\right) }{\eta }-2\int _{x^{\prime }}^{x^{\prime \prime }}\frac{x}{\eta } dF_{i}\left( x\right) . \end{aligned}$$

Since $x^{\prime }\left( F_{i}\left( x^{\prime \prime }\right) -F_{i}\left( x^{\prime }\right) \right) \le \int _{x^{\prime }}^{x^{\prime \prime }} xdF_{i}\left( x\right) \le x^{\prime \prime }\left( F_{i}\left( x^{\prime \prime }\right) -F_{i}\left( x^{\prime }\right) \right) ,$ there exists $\xi \in \left[ x^{\prime },x^{\prime \prime }\right]$ such that

$$ \int _{x^{\prime }}^{x^{\prime \prime }}xdF_{i}\left( x\right) =\xi \left( F_{i}\left( x^{\prime \prime }\right) -F_{i}\left( x^{\prime }\right) \right) . $$

Thus, we have

$$ \left( \Psi +1\right) \varepsilon -2x^{\prime \prime }F_{i}\left( x^{\prime \prime }\right) +2x^{\prime }F_{i}\left( x^{\prime }\right) =2\xi \left( F_{i}\left( x^{\prime \prime }\right) -F_{i}\left( x^{\prime }\right) \right) . $$

Rearranging, we get

$$ \frac{F_{i}\left( x^{\prime \prime }\right) -F_{i}\left( x^{\prime }\right) }{\varepsilon }=\frac{\Psi +1-2F_{i}\left( x^{\prime \prime }\right) }{2\left( x^{\prime }+\xi \right) }. $$

As $\varepsilon \rightarrow 0,$ the right-hand side converges to $\left( \Psi +1-2F_{i}\left( x^{\prime }\right) \right) /\left( 4x^{\prime }\right) .$ Thus, the derivative of $F_{i}$ at $x^{\prime }$ exists and satisfies

$$ f_{i}\left( x^{\prime }\right) =\frac{\Psi +1-2F_{i}\left( x^{\prime }\right) }{4x^{\prime }}, $$

which can be rewritten as (4). $\square$

### Proof of Proposition 2

Suppose $\Psi >1.$ As argued in the main text, only one of the two candidates can place an atom at 1. Otherwise, a candidate can move the mass slightly to the left of 1 and receives a higher payoff. Suppose candidate *i* does not place an atom at 1 in equilibrium. Solving the differential equation (4) with the boundary condition $F_{i}\left( 1\right) =1,$ we have

$$ F_{i}\left( x\right) =\frac{\Psi +1}{2}-\frac{\Psi -1}{2}\frac{1}{\sqrt{x}} $$

on $\left[ \underline{x}^{*},1\right] ,$ where $\underline{x}^{*}\in \left( 0,1\right)$ is obtained by the condition $F_{i}\left( \underline{x}^{*}\right) =0.$ Solving the differential equation

$$ 2xf_{j}\left( x\right) +F_{j}\left( x\right) =\frac{\Psi +1}{2} $$

with the boundary condition $F_{j}\left( \underline{x}^{*}\right) =0,$ we further find that for all $x\in \left[ -1,1\right] ,$ $F_{j}\left( x\right) =F_{i}\left( x\right) .$ The $\Psi <-1$ case is similar. $\square$

### Proof of Proposition 3

Differentiating (5) and (7), we have

$$ \frac{\partial F\left( x\right) }{\partial \Psi }=\left\{ \begin{array}{cc} \frac{1}{2}\left( 1-\frac{1}{\sqrt{x}}\right) & \quad \,{\text {if }}\,\Psi>1\,{\text { and }}\,x>\underline{x}^{*},\\ \frac{1}{2}\left( 1-\frac{1}{\sqrt{-x}}\right) & \quad \,{\text {if }}\,\Psi<-1\,{\text { and }}\,x<\overline{x}^{*}, \end{array} \right. $$

which is smaller than 0. $\square$

### Proof of Proposition 4

Suppose $\left| \Psi \right| \le 1.$ Then, $x_{I}^{*}=x_{O}^{*}=0.$ Thus, $P\left( \Psi \right) ={\mathbb {E}}\left( \left| x_{I}^{*}-x_{O}^{*}\right| \right) =0.$ Suppose $\Psi >1.$ We would like to compute the quantity

$$\begin{aligned}&{\mathbb {E}}\left( \left| x_{I}^{*}-x_{O}^{*}\right| \right) \\&\quad =\int _{\underline{x}^{*}}^{1}\left( \int _{\underline{x}^{*}} ^{1}\left| x-y\right| f\left( x\right) dx\right) f\left( y\right) dy\\&\quad =\int _{\underline{x}^{*}}^{1}\left( \int _{y}^{1}\left( x-y\right) f\left( x\right) dx\right) f\left( y\right) dy+\int _{\underline{x}^{*}}^{1}\left( \int _{\underline{x}^{*}}^{y}\left( y-x\right) f\left( x\right) dx\right) f\left( y\right) dy\\&\quad =2\int _{\underline{x}^{*}}^{1}\left( \int _{y}^{1}\left( x-y\right) f\left( x\right) dx\right) f\left( y\right) dy \end{aligned}$$

Since

$$ f\left( x\right) =\left\{ \begin{array}{cc} 0 & \quad \,{\text {if }}\,x\in \left( -1,\underline{x}^{*}\right) ,\\ \frac{\Psi -1}{4}x^{-\frac{3}{2}} & \quad \,{\text {if }}\,x\in \left( \underline{x}^{*},1\right) , \end{array} \right. $$

we have

$$\begin{aligned}&\int _{y}^{1}\left( x-y\right) f\left( x\right) dx\\&\quad =\frac{\Psi -1}{4}\left( \int _{y}^{1}x^{-\frac{1}{2}}dx-y\int _{y} ^{1}x^{-\frac{3}{2}}dx\right) \\&\quad =\frac{\Psi -1}{4}\left( \left[ 2x^{\frac{1}{2}}\right] _{y}^{1}-y\left[ -2x^{-\frac{1}{2}}\right] _{y}^{1}\right) \\&\quad =\frac{\Psi -1}{4}\left( 2-2y^{\frac{1}{2}}-y\left( 2y^{-\frac{1}{2} }-2\right) \right) \\&\quad =\frac{\Psi -1}{2}\left( 1-2y^{\frac{1}{2}}+y\right) . \end{aligned}$$

Thus,

$$\begin{aligned}&2\int _{\underline{x}^{*}}^{1}\left( \int _{y}^{1}\left( x-y\right) f\left( x\right) dx\right) f\left( y\right) dy\\&\quad =\frac{\Psi -1}{2}\int _{\underline{x}^{*}}^{1}\frac{\Psi -1}{2} \left( 1-2y^{\frac{1}{2}}+y\right) y^{-\frac{3}{2} }dy\\&\quad =\frac{\left( \Psi -1\right) ^{2}}{4}\int _{\underline{x}^{*}} ^{1}\left( y^{-\frac{3}{2}}-2y^{-1}+y^{-\frac{1}{2}}\right) dy\\&\quad =\frac{\left( \Psi -1\right) ^{2}}{4}\left( [-2y^{-\frac{1}{2} }]_{\underline{x}^{*}}^{1}-2\left[ \log \left( y\right) \right] _{\underline{x}^{*}}^{1}+[2y^{\frac{1}{2}}]_{\underline{x}^{*}} ^{1}\right) \\&\quad =\frac{\left( \Psi -1\right) ^{2}}{2}\left( \frac{\Psi +1}{\Psi -1} -1+\log \left( \frac{\Psi -1}{\Psi +1}\right) ^{2}+1-\left( \frac{\Psi -1}{\Psi +1}\right) \right) \\&\quad =\left( \Psi -1\right) ^{2}\log \left( \frac{\Psi -1}{\Psi +1}\right) +2\Psi \frac{\Psi -1}{\Psi +1}. \end{aligned}$$

The expression for $\Psi <-1$ can be obtained similarly. Next,

$$\begin{aligned}&\lim _{\Psi \downarrow 1}P\left( \Psi \right) \\&\quad =\lim _{\Psi \downarrow 1}\left\{ \left( \Psi -1\right) ^{2}\log \left( \frac{\Psi -1}{\Psi +1}\right) +2\Psi \frac{\Psi -1}{\Psi +1}\right\} \\&\quad =\lim _{\Psi \downarrow 1}\frac{\log \left( \frac{\Psi -1}{\Psi +1}\right) }{\frac{1}{\left( \Psi -1\right) ^{2}}}\\&\quad =\lim _{\Psi \downarrow 1}\frac{\frac{2}{\left( \Psi -1\right) \left( \Psi +1\right) }}{-\frac{2}{\left( \Psi -1\right) ^{3}}}\\&\quad =0, \end{aligned}$$

Moreover,

$$\begin{aligned}&\lim _{\Psi \rightarrow +\infty }P\left( \Psi \right) \\&\quad =\lim _{\Psi \rightarrow +\infty }\left\{ \left( \Psi -1\right) ^{2} \log \left( \frac{\Psi -1}{\Psi +1}\right) +2\Psi \frac{\Psi -1}{\Psi +1}\right\} \\&\quad =\lim _{\Psi \rightarrow +\infty }\frac{\log \left( \frac{\Psi -1}{\Psi +1}\right) +\frac{2\Psi }{\left( \Psi +1\right) \left( \Psi -1\right) } }{\frac{1}{\left( \Psi -1\right) ^{2}}}\\&\quad =\lim _{\Psi \rightarrow +\infty }\frac{-\frac{4}{\left( \Psi ^{2}-1\right) ^{2}}}{-\frac{2}{\left( \Psi -1\right) ^{3}}}\\&\quad =0, \end{aligned}$$

where the third equality follows from L’Hôpital’s rule.

To show that $P\left( \Psi \right)$ is single-peaked for $\Psi \in [1,\infty ),$ note that for $\Psi >1,$

$$\begin{aligned} P^{\prime }\left( \Psi \right)&=\frac{2}{\left( \Psi +1\right) ^{2} }\left( 2\left( \Psi ^{2}+\Psi -1\right) +\left( \Psi -1\right) \left( \Psi +1\right) ^{2}\log \left( \frac{\Psi -1}{\Psi +1}\right) \right) ,\\ P^{\prime \prime }\left( \Psi \right)&=\frac{2}{\left( \Psi +1\right) ^{3}}\left( 2\Psi ^{2}+6\Psi +8+\left( \Psi +1\right) ^{3}\ln \left( \frac{\Psi -1}{\Psi +1}\right) \right) . \end{aligned}$$

Since $\lim _{\Psi \downarrow 1}P^{\prime }\left( \Psi \right) >0$ and $P^{\prime }\left( 3\right) <0,$ $P\left( \Psi \right)$ has at least one local maximum on $\left( 1,3\right) .$ Consider $\Psi ^{*}\in \left( 1,3\right)$ such that $P^{\prime }\left( \Psi ^{*}\right) =0.$ Then,

$$\begin{aligned}&P^{\prime \prime }\left( \Psi ^{*}\right) \\&\quad =\frac{2}{\left( \Psi ^{*}+1\right) ^{3}}\left( 2\Psi ^{*2} +6\Psi ^{*}+8+\left( \Psi ^{*}+1\right) ^{3}\left( -\frac{2\left( \Psi ^{*2}+\Psi ^{*}-1\right) }{\left( \Psi ^{*}-1\right) \left( \Psi ^{*}+1\right) ^{2}}\right) \right) \\&\quad =\frac{4\Psi ^{*}-12}{\left( \Psi ^{*}-1\right) \left( \Psi ^{*}+1\right) ^{3}}\\&\quad <0. \end{aligned}$$

Thus, for all $$\Psi \in \left( 1,\Psi ^{*}\right) ,$$ $$P^{\prime }\left( \Psi \right) >0$$ and for all $$\Psi \in (\Psi ^{*},3],$$ $$P^{\prime }\left( \Psi \right) <0.$$ $$P\left( \Psi \right) $$ is single-peaked on the interval $$\left[ 1,3\right] .$$ Next, suppose there exists $$\Psi ^{**}>3$$ such that $$P^{\prime }\left( \Psi ^{**}\right) =0.$$ Then,

$$ P^{\prime \prime }\left( \Psi ^{**}\right) =\frac{4\Psi ^{**} -12}{\left( \Psi ^{**}-1\right) \left( \Psi ^{**}+1\right) ^{3} }>0. $$

Thus, $$\Psi ^{**}$$ is a local minimum. Moreover, for all $$\Psi >\Psi ^{**},$$ since $$P^{\prime }\left( \Psi \right) $$ must be larger than 0,  we have $$P\left( \Psi \right) >P\left( \Psi ^{**}\right) .$$ Since $$\lim _{\Psi \rightarrow +\infty }P\left( \Psi \right) =0$$ and $$P\left( \Psi ^{**}\right) >0,$$ this is a contradiction. This means that for all $$\Psi >3,$$ $$P^{\prime }\left( \Psi \right) <0.$$ $$P\left( \Psi \right) $$ is single-peaked for $$\Psi \in [1,\infty ).$$ $$\square $$

### Proof of Proposition 5

Given the conjectured equilibrium, it is clear that any policy $$x<\underline{x}^{**}$$ results in a lower payoff than $$\underline{x}^{**}.$$ Let $$\Pi _{I}\left( x\right) $$ and $$\Pi _{O}\left( x\right) $$ be the expected payoff of the incumbent *I* and the expected payoff of the opposition *O*,  respectively. The incumbent’s expected payoff of choosing $$x^{\prime }\in \left[ \underline{x}^{**},1\right] $$ is given by

$$\begin{aligned} \Pi _{I}\left( x^{\prime }\right) ={\!}\frac{1}{2}+\int _{-1}^{x^{\prime }}\left( \frac{-x^{\prime }-x{\!}+{\!}\Psi \left( x^{\prime }-x\right) }{\eta }\right) f_{O}\left( x\right) dx{\!}+{\!}\int _{x^{\prime }}^{1}\left( \frac{x^{\prime }+x}{\eta }\right) f_{O}\left( x\right) dx. \end{aligned}$$

Thus,

$$\begin{aligned} \Pi _{I}^{\prime }\left( x^{\prime }\right)= &  \frac{\Psi -1}{\eta }F_{O}\left( x^{\prime }\right) \\ &  -\frac{4x^{\prime }}{\eta }f_{O}\left( x^{\prime }\right) +\frac{1}{\eta }\left( 1-F_{O}\left( x^{\prime }\right) \right) . \end{aligned}$$

Since the distribution $F_{O}\left( x\right)$ given by (11) solves the differential equation

15 $$\begin{aligned} 4xf_{O}\left( x\right) +\left( 2-\Psi \right) F_{O}\left( x\right) =1 \end{aligned}$$

on $$\left( \underline{x}^{**},1\right) $$ and has no atom at $$\underline{x}^{**}$$ and 1,  the incumbent is indifferent between all $$x\in \left[ \underline{x}^{**},1\right] .$$ Likewise, the opposition’s expected payoff of choosing $$x^{\prime }\in [\underline{x}^{**},1)$$ is given by

$$\begin{aligned} \Pi _{O}\left( x^{\prime }\right)&=\frac{1}{2}-\int _{-1}^{x^{\prime }}\left( \frac{x^{\prime }+x}{\eta }\right) f_{I}\left( x\right) dx+\int _{x^{\prime }}^{1}\left( \frac{x^{\prime }+x+\Psi \left( x^{\prime }-x\right) }{\eta }\right) f_{I}\left( x\right) dx \\&\quad +\left( \frac{x^{\prime }+1+\Psi \left( x^{\prime }-1\right) }{\eta }\right) A_{I}. \end{aligned}$$

Thus,

$$ \Pi _{O}^{\prime }\left( x^{\prime }\right) =-\frac{4x^{\prime }}{\eta } f_{I}\left( x^{\prime }\right) -\frac{1}{\eta }F_{I}\left( x^{\prime }\right) +\left( \frac{1+\Psi }{\eta }\right) \left( 1-F_{I}\left( x^{\prime }\right) \right) . $$

Since the distribution $F_{I}\left( x\right)$ given by (10) solves the differential equation

16 $$\begin{aligned} 4xf_{I}\left( x\right) +\left( 2+\Psi \right) F_{I}\left( x\right) =1+\Psi \end{aligned}$$

on $$\left( \underline{x}^{**},1\right) $$ and has no atom at $$\underline{x}^{**},$$ the opposition is indifferent between all $$x\in [\underline{x}^{**},1) .$$ Finally, since the incumbent places an atom at 1,  $$\Pi _{O}\left( x\right) $$ has a downward jump at 1. Thus, choosing 1 is not optimal for the opposition. $$\square $$

### Proof of Proposition 6

Since $$[\underline{x}^{**},1]$$ is the support set of both functions and there is no atom at $$\underline{x}^{**},$$ we must have $$F_{I}\left( \underline{x} ^{**}\right) =F_{O}\left( \underline{x}^{**}\right) =0$$ and $$F_{I}\left( 1\right) =F_{O}\left( 1\right) =1.$$ By (15) and (16),

$$ f_{I}\left( \underline{x}^{**}\right) =\frac{1+\Psi }{4\underline{x}^{**}}>\frac{1}{4\underline{x}^{**}}=f_{O}\left( \underline{x}^{**}\right) . $$

Thus, we must have $$F_{I}\left( x\right) >F_{O}\left( x\right) $$ for *x* larger than but close enough to $$\underline{x}^{**}.$$ Similarly, since $$F_{I}$$ has an atom at 1 but $$F_{O}$$ does not, $$F_{I}\left( 1\right) =F_{O}\left( 1\right) =1$$ implies that $$F_{I}\left( x\right) <F_{O}\left( x\right) $$ for *x* smaller than but close enough to 1. Continuity of $$F_{I} $$ and $$F_{O}$$ implies that there exists $$\hat{x}\in \left( \underline{x} ^{**},1\right) $$ such that $$F_{I}\left( \hat{x}\right) =F_{O}\left( \hat{x}\right) .$$ By (15) and (16 ), we have

$$ 4\hat{x}\left( f_{O}\left( \hat{x}\right) -f_{I}\left( \hat{x}\right) \right) =-\Psi \left( 1-2F_{O}\left( \hat{x}\right) \right) . $$

Let $$\check{x}$$ be such that $$F_{O}\left( \check{x}\right) =\frac{1}{2}.$$ If $$\hat{x}\in \left( 0,\check{x}\right) ,$$ then $$f_{O}\left( \hat{x}\right) <f_{I}\left( \hat{x}\right) ,$$ which means that $$F_{I}$$ crosses $$F_{O}$$ from below. Since $$F_{I}\left( x\right) >F_{O}\left( x\right) $$ for *x* larger than but close enough to $$\underline{x}^{**},$$ this is impossible. If $$\hat{x}=\check{x},$$ then $$f_{O}\left( \hat{x}\right) =f_{I}\left( \hat{x}\right) .$$ Differentiate (15) and (16), we have

$$ 2\hat{x}\left( f_{O}^{\prime }\left( \hat{x}\right) -f_{I}^{\prime }\left( \hat{x}\right) \right) =\Psi f_{O}\left( \hat{x}\right) >0, $$

which means that $F_{I}$ touches $F_{O}$ from below. This is again impossible. If $\hat{x}\in \left( \check{x},1\right) ,$ then $f_{O}\left( \hat{x}\right) >f_{I}\left( \hat{x}\right) ,$ which means that $F_{I}$ crosses $F_{O}$ from above. This means that $F_{I}$ and $F_{O}$ cross only once. Thus, $\hat{x}$ is unique and satisfies $F_{I}\left( \hat{x}\right) =F_{O}\left( \hat{x}\right) >\frac{1}{2}.$ $\square$

### Proof of Proposition 7

By Proposition 5, we have, for all $$x\in \left( \underline{x}^{**},1\right) ,$$

$$\begin{aligned} f_{O}\left( x\right)&=\frac{\Psi -1}{4}x^{-\frac{6-\Psi }{4}}\\ f_{I}\left( x\right)&=\left\{ \begin{array}{cc} \frac{1+\Psi }{4}\left( \Psi -1\right) ^{\frac{2+\Psi }{2-\Psi }}x^{-\frac{6+\Psi }{4}} & \quad \,{\text {if }}\,\Psi \ne 2,\\ \frac{3}{4}e^{-4}x^{-2} & \quad \,{\text {if }}\,\Psi =2. \end{array} \right. \end{aligned}$$

Moreover,

$$ A_{I}=\left\{ \begin{array}{cc} 1-\frac{1+\Psi }{2+\Psi }\left( 1-\left( \Psi -1\right) ^{\frac{2+\Psi }{2-\Psi }}\right) & \quad \,{\text {if }}\,\Psi \ne 2,\\ \frac{1}{4}+\frac{3}{4}e^{-4} & \quad \,{\text {if }}\,\Psi =2. \end{array} \right. $$

We would like to calculate

17 $$\begin{aligned}&{\mathbb {E}}\left( \left| x_{I}^{*}-x_{O}^{*}\right| \right) \nonumber \\&\quad =\int _{\underline{x}^{**}}^{1}\left( \int _{\underline{x}^{**} }^{1}\left| y-x\right| f_{O}\left( x\right) dx\right) f_{I}\left( y\right) dy+\left( \int _{\underline{x}^{**}}^{1}\left( 1-x\right) f_{O}\left( x\right) dx\right) A_{I}\nonumber \\&\quad =\int _{\underline{x}^{**}}^{1}\left( \int _{y}^{1}\left( x-y\right) f_{O}\left( x\right) dx+\int _{\underline{x}^{**}}^{y}\left( y-x\right) f_{O}\left( x\right) dx\right) f_{I}\left( y\right) dy\nonumber \\&\qquad +\left( \int _{\underline{x}^{**}}^{1}\left( 1-x\right) f_{O}\left( x\right) dx\right) A_{I} . \end{aligned}$$

For $\Psi \in \left( 1,\infty \right) \backslash \left\{ 2\right\} ,$ the first term of the inner integral of the first term of (17) is

$$\begin{aligned}&\int _{y}^{1}\left( x-y\right) f_{O}\left( x\right) dx\\&\quad =\frac{\Psi -1}{4}\int _{y}^{1}\left( x^{-\frac{2-\Psi }{4}}-yx^{-\frac{6-\Psi }{4}}\right) dx\\&\quad =\frac{\Psi -1}{4}\frac{4}{2+\Psi }\left[ x^{\frac{2+\Psi }{4}}\right] _{y}^{1}-\frac{\Psi -1}{4}\left( -\frac{4}{2-\Psi }\right) y\left[ x^{-\frac{2-\Psi }{4}}\right] _{y}^{1}\\&\quad =\frac{\Psi -1}{2+\Psi }\left( 1-y^{\frac{2+\Psi }{4}}\right) +\frac{\Psi -1}{2-\Psi }\left( y-y^{\frac{2+\Psi }{4}}\right) \\&\quad =\frac{\Psi -1}{2+\Psi }-\frac{\Psi -1}{\Psi -2}y+4\frac{\Psi -1}{\Psi ^{2}-4}y^{\frac{2+\Psi }{4}}. \end{aligned}$$

The second term of the inner integral of the first term of (17) is

$$\begin{aligned}&\int _{\underline{x}^{**}}^{y}\left( y-x\right) f_{O}\left( x\right) dx\\&\quad =\frac{\Psi -1}{4}\int _{\underline{x}^{**}}^{y}\left( yx^{-\frac{6-\Psi }{4}}-x^{-\frac{2-\Psi }{4}}\right) dx\\&\quad =\frac{\Psi -1}{4}\left( -\frac{4}{2-\Psi }\right) y\left[ x^{-\frac{2-\Psi }{4}}\right] _{\underline{x}^{**}}^{y}-\frac{\Psi -1}{4}\frac{4}{2+\Psi }\left[ x^{\frac{2+\Psi }{4}}\right] _{\underline{x}^{**} }^{y}\\&\quad =\frac{\Psi -1}{\Psi -2}y\left[ x^{-\frac{2-\Psi }{4}}\right] _{\underline{x}^{**}}^{y}-\frac{\Psi -1}{2+\Psi }\left[ x^{\frac{2+\Psi }{4}}\right] _{\underline{x}^{**}}^{y}\\&\quad =\frac{\Psi -1}{\Psi -2}\left( y^{\frac{2+\Psi }{4}}-y\left( \Psi -1\right) ^{-1}\right) -\frac{\Psi -1}{2+\Psi }\left( y^{\frac{2+\Psi }{4}}-\left( \Psi -1\right) ^{\frac{2+\Psi }{2-\Psi }}\right) \\&\quad =4\frac{\Psi -1}{\Psi ^{2}-4}y^{\frac{2+\Psi }{4}}-\frac{1}{\Psi -2}y+\frac{\left( \Psi -1\right) ^{\frac{4}{2-\Psi }}}{2+\Psi }. \end{aligned}$$

Thus, the inner integral of the first term is

$$\begin{aligned}&\int _{y}^{1}\left( x-y\right) f_{O}\left( x\right) dx+\int _{\underline{x}^{**}}^{y}\left( y-x\right) f_{O}\left( x\right) dx\\&\quad =\left( \frac{\Psi -1}{2+\Psi }-\frac{\Psi -1}{\Psi -2}y+ 4\frac{\Psi -1}{\Psi ^{2}-4}y^{\frac{2+\Psi }{4}}\right) \\&\qquad + \left( 4\frac{\Psi -1}{\Psi ^{2}-4}y^{\frac{2+\Psi }{4}}-\frac{1}{\Psi -2}y+\frac{\left( \Psi -1\right) ^{\frac{4}{2-\Psi }}}{2+\Psi }\right) \\&\quad =8\frac{\Psi -1}{\Psi ^{2}-4}y^{\frac{2+\Psi }{4}}-\frac{\Psi }{\Psi -2}y+\frac{\Psi -1}{2+\Psi }+\frac{\left( \Psi -1\right) ^{\frac{4}{2-\Psi }}}{2+\Psi }. \end{aligned}$$

Thus, the first term of (17) becomes

$$\begin{aligned}&\int _{\underline{x}^{**}}^{1}\left( \int _{y}^{1}\left( x-y\right) f_{O}\left( x\right) dx+\int _{\underline{x}^{**}}^{y}\left( y-x\right) f_{O}\left( x\right) dx\right) f_{I}\left( y\right) dy\\&\quad =8\frac{\Psi -1}{\Psi ^{2}-4}\frac{1+\Psi }{4}\left( \Psi -1\right) ^{\frac{2+\Psi }{2-\Psi }}\int _{\underline{x}^{**}} ^{1}y^{\frac{2+\Psi }{4}}y^{-\frac{6+\Psi }{4}}dy\\&\qquad +\left( -\frac{\Psi }{\Psi -2}\right) \frac{1+\Psi }{4}\left( \Psi -1\right) ^{\frac{2+\Psi }{2-\Psi }}\int _{\underline{x}^{**}} ^{1}y^{-\frac{2+\Psi }{4}}dy\\&\qquad +\left( \frac{\Psi -1}{2+\Psi }+\frac{\left( \Psi -1\right) ^{\frac{4}{2-\Psi }}}{2+\Psi }\right) \left( \frac{1+\Psi }{4}\left( \Psi -1\right) ^{\frac{2+\Psi }{2-\Psi }}\int _{\underline{x}^{**}}^{1}y^{-\frac{6+\Psi }{4}}dy\right) \\&\quad =2\frac{\left( 1+\Psi \right) \left( \Psi -1\right) ^{\frac{4}{2-\Psi }} }{\Psi ^{2}-4}\int _{\underline{x}^{**}}^{1}\frac{dy}{y}\\&\qquad -\frac{\Psi }{\Psi -2}\frac{1+\Psi }{4}\left( \Psi -1\right) ^{\frac{2+\Psi }{2-\Psi }}\left( \frac{4}{2-\Psi }\right) \left[ y^{\frac{2-\Psi }{4} }\right] _{\underline{x}^{**}}^{1}\\&\qquad +\left( \frac{\Psi -1}{2+\Psi }+\frac{\left( \Psi -1\right) ^{\frac{4}{2-\Psi }}}{2+\Psi }\right) \left( \frac{1+\Psi }{2+\Psi }\left( 1-\left( \Psi -1\right) ^{\frac{2+\Psi }{2-\Psi }}\right) \right) \\&\quad =\frac{8\left( 1+\Psi \right) \left( \Psi -1\right) ^{\frac{4}{2-\Psi }} }{\left( \Psi -2\right) ^{2}\left( \Psi +2\right) }\log \left( \Psi -1\right) -\frac{\Psi \left( 1+\Psi \right) }{\Psi -2}\left( \Psi -1\right) ^{\frac{2+\Psi }{2-\Psi }}\\&\qquad +\left( \frac{\Psi -1}{2+\Psi }+\frac{\left( \Psi -1\right) ^{\frac{4}{2-\Psi }}}{2+\Psi }\right) \left( \frac{1+\Psi }{2+\Psi }\left( 1-\left( \Psi -1\right) ^{\frac{2+\Psi }{2-\Psi }}\right) \right) . \end{aligned}$$

Finally,

$$\begin{aligned} \int _{\underline{x}^{**}}^{1}\left( 1-x\right) f_{O}\left( x\right) dx&=4\frac{\Psi -1}{\Psi ^{2}-4}-\frac{1}{\Psi -2}+\frac{\left( \Psi -1\right) ^{\frac{4}{2-\Psi }}}{2+\Psi }\\&=\frac{3}{\Psi +2}+\frac{\left( \Psi -1\right) ^{\frac{4}{2-\Psi }}}{2+\Psi }. \end{aligned}$$

The second term of (17) is

$$\begin{aligned}&\left( \int _{\underline{x}^{**}}^{1}\left( 1-x\right) f_{O}\left( x\right) dx\right) A_{I}\\&\quad =\left( \frac{3}{\Psi +2}+\frac{\left( \Psi -1\right) ^{\frac{4}{2-\Psi }} }{2+\Psi }\right) \left( 1-\frac{1+\Psi }{2+\Psi }\left( 1-\left( \Psi -1\right) ^{\frac{2+\Psi }{2-\Psi }}\right) \right) \\&\quad =\left( \frac{3}{\Psi +2}+\frac{\left( \Psi -1\right) ^{\frac{4}{2-\Psi }} }{2+\Psi }\right) \left( \frac{1}{2+\Psi }+\frac{1+\Psi }{2+\Psi }\left( \Psi -1\right) ^{\frac{2+\Psi }{2-\Psi }}\right) \\&\quad =\frac{3}{\left( \Psi +2\right) ^{2}}+\frac{\left( \Psi -1\right) ^{\frac{4}{2-\Psi }}}{\left( 2+\Psi \right) ^{2}}+3\frac{1+\Psi }{\left( 2+\Psi \right) ^{2}}\left( \Psi -1\right) ^{\frac{2+\Psi }{2-\Psi }} +\frac{1+\Psi }{\left( 2+\Psi \right) ^{2}}\left( \Psi -1\right) ^{\frac{6+\Psi }{2-\Psi }}. \end{aligned}$$

Summing them up, we have

$$\begin{aligned} {\mathbb {E}}\left( \left| x_{I}^{*}-x_{O}^{*}\right| \right) =\,&\frac{8\left( \Psi +1\right) \left( \Psi -1\right) ^{\frac{4}{2-\Psi }} }{\left( \Psi -2\right) ^{2}\left( \Psi +2\right) }\log \left( \Psi -1\right) \\&+\frac{\Psi ^{2}+2}{\left( \Psi +2\right) ^{2}}-\frac{\Psi ^{3}+5\Psi ^{2}+7\Psi +6}{\left( \Psi -2\right) \left( \Psi +2\right) ^{2} }\left( \Psi -1\right) ^{\frac{4}{2-\Psi }}\\&-\frac{\Psi ^{3}+2\Psi ^{2}+11\Psi +10}{\left( \Psi -2\right) \left( \Psi +2\right) ^{2}}\left( \Psi -1\right) ^{\frac{2+\Psi }{2-\Psi }}. \end{aligned}$$

Moreover,

$$ \lim _{\Psi \downarrow 1}{\mathbb {E}}\left( \left| x_{I}^{*}-x_{O}^{*}\right| \right) =\frac{1}{3} $$

and

$$ \lim _{\Psi \rightarrow +\infty }{\mathbb {E}}\left( \left| x_{I}^{*} -x_{O}^{*}\right| \right) =0. $$

The latter follows from the fact that

$$ \lim _{\Psi \rightarrow +\infty }\underline{x}^{**}=1, $$

which follows from

$$\begin{aligned}&\lim _{\Psi \rightarrow +\infty }\log \left( \underline{x}^{**}\right) \\&\quad =\lim _{\Psi \rightarrow +\infty }\frac{4\log \left( \Psi -1\right) }{2-\Psi }\\&\quad =\lim _{\Psi \rightarrow +\infty }\frac{4\frac{1}{\Psi -1}}{-1}\\&\quad =0. \end{aligned}$$

We can further calculate (17) for $\Psi =2.$ We have

$$\begin{aligned}&\int _{y}^{1}\left( x-y\right) f_{O}\left( x\right) dx\\&\quad =\frac{1}{4}\int _{y}^{1}\left( 1-yx^{-1}\right) dx\\&\quad =\frac{1}{4}\left( 1-y\right) -\frac{1}{4}y\left[ \log \left( x\right) \right] _{y}^{1}\\&\quad =\frac{1}{4}\left( 1-y+y\log y\right) \end{aligned}$$

and

$$\begin{aligned}&\int _{\underline{x}^{**}}^{y}\left( y-x\right) f_{O}\left( x\right) dx\\&\quad =\frac{1}{4}\int _{\underline{x}^{**}}^{y}\left( yx^{-1}-1\right) dx\\&\quad =\frac{1}{4}\left( \left[ y\log x\right] _{\underline{x}^{**}} ^{y}-y+\underline{x}^{**}\right) \\&\quad =\frac{1}{4}\left( 3y+e^{-4}+y\log y\right) . \end{aligned}$$

Then, we have

$$\begin{aligned}&\int _{\underline{x}^{**}}^{1}\left( \int _{y}^{1}\left( x-y\right) f_{O}\left( x\right) dx+\int _{\underline{x}^{**}}^{y}\left( y-x\right) f_{O}\left( x\right) dx\right) f_{I}\left( y\right) dy\\&\quad =\frac{3}{4}e^{-4}\int _{\underline{x}^{**}}^{1}\frac{1}{4}\left( 1+e^{-4}+2y+2y\log y\right) y^{-2}dy\\&\quad =\frac{3}{16}e^{-4}\int _{\underline{x}^{**}}^{1}\left( \left( 1+e^{-4}\right) y^{-2}+2y^{-1}+2y^{-1}\log y\right) dy\\&\quad =\frac{3}{16}e^{-4}\left( \left( 1+e^{-4}\right) \left[ -y^{-1}\right] _{\underline{x}^{**}}^{1}+2\left[ \log y\right] _{\underline{x} ^{**}}^{1}+[\left( \log y\right) ^{2}]_{\underline{x}^{**}} ^{1}\right) \\&\quad =\frac{3}{16}e^{-4}\left( \left( 1+e^{-4}\right) \left( e^{4}-1\right) +2\left( 4\right) -\left( -4\right) ^{2}\right) \\&\quad =\frac{3}{16}\left( 1-e^{-8}-8e^{-4}\right) . \end{aligned}$$

Finally,

$$\begin{aligned}&\left( \int _{\underline{x}^{**}}^{1}\left( 1-x\right) f_{O}\left( x\right) dx\right) A_{I}\\&\quad =\frac{1}{4}\left( 3+e^{-4}\right) \left( \frac{1}{4} +\frac{3}{4}e^{-4}\right) \\&\quad =\frac{1}{16}\left( 3e^{-4}+1\right) \left( e^{-4}+3\right) . \end{aligned}$$

Summing them up, we have

$$ P\left( 2\right) =\frac{3}{16}\left( 1-e^{-8}-8e^{-4}\right) +\frac{1}{16}\left( 3e^{-4}+1\right) \left( e^{-4}+3\right) =\frac{3}{8}-\frac{7}{8}e^{-4}. $$

$$\square $$

## Footnotes

1 This point, however, is debated. See Abramowitz and Fiorina (2013), Fiorina et al. (2010) and Fiorina (2016).

2 A more thorough review of this literature is given in Sect. 1.1.

3 “Informed” and “uninformed” voters are standard terminologies of the literature (Baron 1994; Grossman and Helpman 1996). Uninformed voters are sometimes also referred to as “impressionable” voters.

4 From a public choice perspective, this essentially means that campaign contributions create a Condorcet cycle and, as a result, a Condorcet winner no longer exists.

5 For example, using Calvert (1985)’s model, Fiorina (1999) argues that the proliferation of polling and other means of two-way communication implies that voter preference uncertainty is likely to have reduced and thus unlikely to be a source for increasing policy divergence. In our model, reducing voter uncertainty increases the donor’s incentive to influence the election and could increase policy polarization.

6 See Sections 3.5 and 7.4 of Persson and Tabellini (2002).

7 In other words, the most commonly used approach in the literature makes use of what Duggan (2017) calls the “stochastic partisanship” model and our model is what he calls a “stochastic preference” model. It has been noted in other contexts that the two models can lead to radically different conclusions. In a Downsian model where the candidates first choose the policies and then invest in costly valences, Ashworth and de Mesquita (2009) find a similar result that polarization occurs in the “stochastic preference” model, but not in the “stochastic partisanship” model. The equilibrium and the mechanism behind polarization in our model are, however, very different from theirs.

8 Venkatesh (2020) likewise restricts his attention to such a symmetric setting with two opposite activists in his study of political activism. Instead of attracting uninformed voters, he assumes that an activist’s support increases the valence of a candidate and finds similarly that policy polarization decreases in the presence of political activism.

9 The quadratic formulation of the donor’s payoff is also used by Persson and Tabellini (2002).

10 Thus, we have assumed that the donor has an extreme preference. Empirically, it has been found that citizens actively engaged in politics are more ideologically extreme and polarized than the average constituents (Abramowitz 2010). Barber (2016), McElwee (2016), McElwee et al. (2016) report polarization of donors’ ideological positions at presidential, senate, and mayoral levels. In a related model with heterogeneous donors, Hirata and Kamada (2020) show that only the most extreme donors would contribute. Even though their result depends on the functional form of the donors’ utility functions, it suggests that generally citizens and interest groups with extreme preferences are more likely to donate, as they have more to lose.

11 See Aragonès and Ponsatí (2022) for a discussion and summary of empirical evidence for exogenous shocks that induce changes in voters’ policy preferences.

12 An alternative assumption is that the donor’s contribution is used to ‘flip’ existing votes through vote buying or intimidation. The corresponding assumption in this case is that the cost of vote flipping is proportional to the number of flipped votes. This alternative formulation does not require the existence of uninformed voters and is perhaps more descriptive of elections in authoritarian regimes.

13 Formally, $N_{y}\left( x_{I},x_{O}\right)$ is defined by

$$ N_{y}\left( x_{I},x_{O}\right) :=\left( \underline{\textbf{y}}\left( x_{I},x_{O}\right) ,\overline{\textbf{y}}\left( x_{I},x_{O}\right) \right) , $$

where

$$ \overline{\textbf{y}}\left( x_{I},x_{O}\right) :=\left\{ \begin{array}{ll} x_{I}+x_{O}+\frac{\eta }{2} & \quad \,{\text {if }}\,x_{I}>x_{O},\\ -\left( x_{I}+x_{O}\right) +\frac{\eta }{2} & \quad \,{\text {if }}\,x_{I}<x_{O}, \end{array} \right. $$

and

$$ \underline{\textbf{y}}\left( x_{I},x_{O}\right) :=\left\{ \begin{array}{cc} x_{I}+x_{O}-\frac{\eta }{2} & \quad \,{\text {if }}\,x_{I}>x_{O},\\ -\left( x_{I}+x_{O}\right) -\frac{\eta }{2} & \quad \,{\text {if }}\,x_{I}<x_{O}. \end{array} \right. $$

14 A comprehensive overview of the literature on the median voter theorem is available in Duggan (2017).

15 Consider the simple case when the two positions are larger than $x_{j}^{*}.$ In this case, the first expression in (3) applies. Since the winning probability is strictly increasing in $x_{i},$ candidate *i* prefers the larger point. More generally, when candidate *j* uses a mixed strategy, the first expression applies for some $x_{j}$’s and the second expression for others. As long as candidate *j* does not choose any point between the points, it remains the case that candidate *i* prefers the larger point.

16 Note that $P\left( \Psi \right)$ is continuous at $\Psi =2,$ where $P\left( 2\right) =\frac{3}{8}-\frac{7}{8}e^{-4}.$

17 Due to the complexity of the expression (13), we have not been able to show this analytically as in the case of (9). However, numerical illustration of Fig. 3 shows that this is indeed the case.

18 Due to the complexity of the expressions (9) and (13), we have not shown this result analytically. However, since (9) and (13) are functions of a single variable, this can be verified numerically easily, as illustrated in Fig. 3.

19 This result therefore has no counterpart in Ball (1999) or Dunaway and Munoz-Garcia (2020), as it is impossible for a candidate’s policy to move away from the other candidate’s policy in both directions in a pure strategy equilibrium.

20 See Fiorina (2016), Figure 1. However, according to a report produced by Issue One, a dozen megadonors accounts for 7.5 percent of political giving between 2009 and 2020. It is thus reasonable to assume that campaign contributions remain dominated by a relatively small number of extreme donors. See https://www.issueone.org/wp-content/uploads/2021/04/Issue-One-Outsized-Influence-Report-final.pdf.

21 For example, Brock (2021) documents the gradual breakdown of bipartisanship in the House and Senate Agriculture committees from 2009 to 2019 and the increase in agribusiness lobbying associated with it.
